# Sustainability in large language model supply chains-insights and recommendations using analysis of utility for affecting factors

**DOI:** 10.1038/s41598-025-17937-8

**Published:** 2025-09-29

**Authors:** Vinaytosh Mishra, Deepika Saxena, Kishu Gupta, Sakshi Patni, Ashutosh Kumar Singh

**Affiliations:** 1 Datta Meghe Institute of Higher Education and Research, Wardha, Maharashtra 442107 India; 2 Thumbay College of Management and AI in Healthcare, Gulf Medical University, Ajman, UAE; 3 School of Computer Science and Engineering, The University of Aizu, Aizuwakamatsu, 965-0006 Japan; 4 Department of Computer Science and Engineering, National Sun Yat-sen University, Kaohsiung, 804 Taiwan; 5 Department of Computer Applications, Panipat Institute of Engineering and Technology, Panipat, Haryana India; 6 Department of Computer Science and Engineering, Indian Institute of Information Technology Bhopal, Bhopal, 462003 India

**Keywords:** LLMs, Supply Chain, Sustainability, Conjoint Analysis, Environmental impact, Information technology

## Abstract

The increasing adoption of Large Language Models (LLMs) has intensified concerns regarding the sustainability of their supply chains, particularly concerning energy consumption, resource utilization, and carbon emissions. To address these concerns, this study proposes a two-step approach. First, a Delphi method is employed to systematically identify the critical factors affecting the sustainability of LLM supply chains. Expert consensus through four rounds of feedback highlights key factors such as Environmental Impact, Computational Efficiency & Resource Optimization, Data Quality & Ethical Considerations, and Social Responsibility & Governance. In the second step, the identified factor’s relative importance was calculated using Conjoint Analysis, a statistical technique used to determine how respondents value different factors of a supply chain of LLMs. This prioritization helps formulate strategies to make LLM’s supply chain sustainable. The low score for environmental impact suggests a lack of awareness about the sustainability of LLMs’ supply chain. The study finds Data Quality and Ethical considerations to be the most important considerations for the respondents. Thus, it provides a framework for implementing sustainable practices in LLMs’ supply chains in resource-constrained settings. The results demonstrate the effectiveness of this combined Delphi-Conjoint Analysis approach, providing actionable insights for AI organizations aiming to enhance the sustainability of their LLM operations.

## Introduction

The world has seen unprecedented interest in Large Language Models (LLMs) in the last few years. The ubiquitous purpose of LLMs is to improve productivity and make the automation of various tasks possible. General purpose LLMs such as ChatGPT4o have around 1.76 trillion parameters, and their training and use require huge computational power, which results in substantial energy consumption. The increasing adoption of LLMs in every industry has raised significant concerns regarding the sustainability of their supply chain, particularly in terms of energy consumption, resource utilization, and carbon emissions. These concerns are preliminary only, and usage will increase further in the coming years as adoption increases. Thus, it becomes imperative that we understand the environmental impact of LLMs for future developments in artificial intelligence (AI) and natural language processing (NLP). The 540-billion parameter Pathways Language Model (PaLM) training using 6144 TPU v4 chips brought to light the enormous computational resources needed for such large-scale models^1^. This degree of resource consumption raises concerns about such operations’ environmental sustainability. Increased carbon emissions may result from the energy-intensive nature of training LLMs, especially if the energy sources are non-renewable^2^. This problem is made worse because many regions rely heavily on fossil fuels for energy. Hence, it is critical to investigate more environmentally friendly methods for training and deploying models.

Furthermore, the materials and resources used to create LLMs influence the environment and energy usage. Mining and processing raw materials can harm ecosystems and increase pollution, as the production of GPUs and TPUs–hardware required for training these models–involves these activities^2,3^. The evaluation of LLMs should encompass their performance, sustainability, privacy, digital divide, and ethical implications^2^. This holistic perspective is essential for developing responsible AI technologies that minimize environmental harm. The academic world is gung-ho about the capabilities of LLMs, and there is a scarcity of literature deliberating on the sustainability of the supply chain of these models. This research attempts to fill this research gap.

While the technical advancements and capabilities of LLMs are well-documented, particularly their role in revolutionizing language tasks through transformer-based architectures, there remains a significant gap in understanding their broader systemic impact. What we know includes the high energy consumption during training, the carbon footprint of large-scale GPU/TPU usage, and ethical concerns related to data quality and governance. However, what we don’t know is how these diverse concerns interact across the LLM supply chain from data collection to deployment and which factors stakeholders consider most critical in ensuring sustainability. Moreover, there is limited empirical evidence quantifying the relative importance of environmental versus ethical or computational considerations, especially in resource-constrained regions. This study attempts to bridge that gap by identifying and prioritizing the key factors affecting the sustainability of LLM supply chains using a combined Delphi-Conjoint Analysis framework. The supply chain of LLMs is still evolving, and other factors may be affecting its sustainability. This study uses expert opinion to identify factors affecting the sustainability of LLMs’ supply chains and group them into coherent groups. These groups were further analyzed to determine the utility of stakeholders for these coherent groups. With this background, this study has two objectives:

- Identify the factors affecting the sustainability of LLM’s supply chain.
- Find out the relative importance of these factors for allocating the resources.

The next section reviews the related literature to identify factors affecting the sustainability of the LLM supply chain. The third section discusses the approach to identifying and prioritizing factors affecting sustainability. The fourth section discusses the Results, followed by a discussion and concluding section that discusses the implications and future directions of the research.

## Related work

LLMs are a notable development in Natural Language Processing (NLP) because of their exceptional capacity for generating and comprehending human language^4^. The development of LLMs can be linked to previous computational techniques that were less able to manage the complexity of human language, such as rule-based systems and statistical models^5^. A significant turning point in this field was the advent of deep learning techniques, especially transformer architecture, which made it possible to create models that could learn from enormous volumes of text data without requiring task-specific training^6^. The transformer architecture enables models to process and generate text by focusing on the relationships between words in a sentence rather than relying on sequential processing as in previous models^7^. This capability has led to the creation of powerful models like the Generative Pre-trained Transformer (GPT) series, which have set new benchmarks in various NLP tasks, including text generation, translation, and summarization^8,9^. In-context learning, where models can perform tasks based on a few examples provided during inference, further distinguishes LLMs from their predecessors, allowing for greater flexibility and adaptability in real-world applications^10^. GPT and Gemini rely on the Transformer architecture, enabling parameters and model size scaling. Starting with GPT-4 and Gemini, there is a strong focus on multi-modal AI, where models are text-based and capable of processing and generating images, videos, and other modalities. As the number of parameters increases, these models have demonstrated better generalization across tasks, requiring fewer task-specific instructions or fine-tuning. Table 1 summarize the historical development of transformer architecture-based LLMs^11–15^.

**Table 1:** Evolution of transformer-based large language models and their impact.

Model	Year	Parameters	Key features	Impact
GPT-1	2018	117 million	Transformer architecture, unsupervised pretraining, fine-tuning for specific tasks	Showed that pretraining on large text corpora transfers well to downstream tasks
GPT-1	2018	117 million	Transformer architecture, unsupervised pretraining, fine-tuning for specific tasks	It showed that pretraining on large text corpora transfers well to downstream tasks
BERT	2018	110 million (base) / 340 million (large)	Bidirectional transformers, pretraining with masked language modeling	Revolutionized NLP benchmarks; led to wide adoption in classification and QA tasks
GPT-2	2019	1.5 billion	Larger model size, text generation, strong performance without task-specific training	Demonstrated high-quality text generation and raised concerns about potential misuse
GPT-3	2020	175 billion	Few-shot learning, general-purpose text generation across multiple tasks	Popularized the use of large-scale language models for diverse AI applications
PALM	2022	540 billion	Dense decoder-only transformer, chain-of-thought prompting	Achieved strong reasoning and arithmetic performance; precursor to PaLM-E and PaLM-2
GPT-4	2023	Estimated 100+ billion to trillions	Multimodal capabilities (text and images), improved reasoning	Enhanced accuracy, multimodal understanding, and problem-solving capabilities
Gemini 1	2023	Estimated 100+ billion	Multimodal AI combining text and vision, inspired by DeepMind’s advancements	Competes with GPT-4, focusing on integrating language and vision for broader AI applications
Gemini 1.5 / 2	Late 2023/Early 2024	Estimated trillions	Continued advancements in multimodal AI, reasoning, and grounding	Expected to push the boundaries of AI performance, particularly in complex reasoning and autonomy
LLaMA	2023	7B–65B	Open-access models optimized for performance on smaller compute	Democratized LLM research by enabling researchers to experiment without massive infrastructure

### Sustainable computing

The energy demands of servers employed in large language models (LLMs) are significant, primarily owing to the extensive computational resources required for training and inference. Large Language Models (LLMs), including GPT and PaLM, include hundreds of billions of parameters, requiring high-capacity processors such as GPUs and substantial cloud infrastructure. This results in considerable energy consumption and greenhouse gas emissions during training and deployment^16^. The complexity of LLMs raises concerns about their cost-effectiveness, as the benefits gained from their advanced capabilities may not justify the escalating energy and financial costs associated with their operation^17^. Moreover, the hardware specifications encompass processor power and factors related to model compression and hardware acceleration methods that enhance performance while reducing energy consumption^18^. Sophisticated hardware solutions, such as Field Programmable Gate Arrays (FPGAs), have been suggested to improve energy efficiency, while difficulties persist in efficiently programming these systems^19^. With the rising demand for LLMs, the sustainability of their deployment is becoming increasingly vital, requiring a balance between performance and environmental effects^17^.

### Supply chain of LLMs

The supply Chain of Large Language models involves various stages such as (1) Data Collection, (2) Data processing, (3) Model Training, (4) Fine Tuning and Testing, (5) Deployment, and (6)Maintenance and Update. The data is collected from various databases, web resources and books^20^. This data is then processed, cleaned, and formatted for training. High-performance computing infrastructure, including GPUs and TPUs, is essential to process and train these models, requiring significant energy and resources. After their training LLMs undergo fine-tuning and testing to enhance their accuracy. Deployment occurs through cloud services or edge devices for accessibility. The concluding phase entails ongoing updates, maintenance, and ethical oversight to guarantee equity, precision, and responsible use^21^.

The computational power for training LLMs uses GPUs, and TPUs require a lot of energy; hence, the carbon footprint is significant. Energy-efficient hardware and renewable energy sources are recommended to train these LLMs^16,17^. Gathering and analyzing extensive volumes of varied data necessitates the assurance of data quality, the reduction of biases, and the responsible management of sensitive information. The ethical acquisition of data and the assurance of equity are essential for sustainable operations^22^. The manufacturing, utilization, and disposal of hardware, including GPUs, TPUs, and servers, can generate electronic waste. The sustainable management of hardware supply chains is crucial for mitigating environmental concerns^23^. Data centres, where massive language models are trained and deployed, frequently necessitate substantial cooling systems that utilize considerable quantities of water. Effective water utilization strategies are essential for sustainability^24^. Collaborating with stakeholders, sharing research on sustainability efforts, and being transparent about resource usage and environmental impact help achieve a sustainable supply chain.

Recent advancements such as GPT-4 and Gemini 1.5 exemplify multi-modal AI, capable of processing and generating content across text, image, and, in some cases, audio domains. These models have demonstrated state-of-the-art performance in tasks like image captioning, code understanding from screenshots, and reasoning across visual-textual inputs. However, this performance comes at a high computational cost, requiring powerful parallel hardware such as TPUs and A100 GPUs and significantly more energy and memory than unimodal models^13,14,16^. This raises critical concerns about the scalability and sustainability of such models, particularly in resource-constrained contexts.

## Methods

**Fig. 1:**
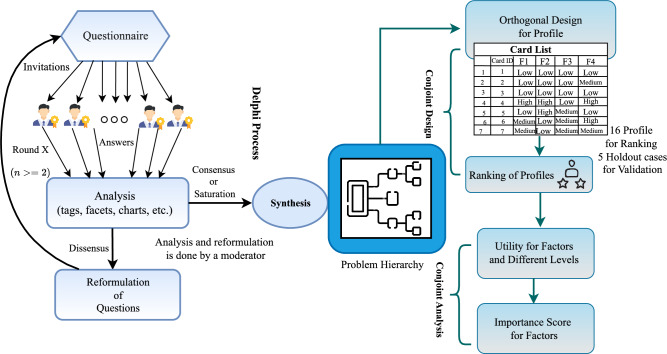
Summary of the Delphi conjoint approach.

This study uses the Delphi method, an iterative process used to gather opinions from a group of experts to reach a consensus. Secondly, this study uses Conjoint Analysis, a statistical technique, to determine how technology experts value different factors identified for the objective. The summary of the model prioritizing the factors affecting sustainability of LLMs supply chain using Delphi-Conjoint Approach is given in the Fig. 1.

### Identification of factors

This study uses a focus group selected based on their knowledge, experience, and expertise to provide meaningful insights. The number of experts N has been decided upon to ensure that data collection and focused discussions are achieved. In the first round, experts were asked open-ended questions about factors affecting the sustainability of the LLMs supply chain. These question includes: what are the key factors affecting the sustainability of Large Language Models’ supply chains? What environmental, ethical, or governance-related challenges do you consider most critical in the context of Large Language Models? These questions were designed to elicit extensive expert insights without constraining responses prematurely, thus ensuring comprehensive and varied input that could be effectively aggregated in subsequent rounds. Let the response from the expert *i* is denoted by $Q_{1i}.$ The aggregation of the response for the round one is given by $A_1.$

1 $$\begin{aligned} A_1=Summary\{Q_{11}, Q_{12}, \ldots , Q_{1N}\} \end{aligned}$$

The aggregated responses are presented again to the panel of experts. This feedback is anonymized to avoid bias, and experts are encouraged to reflect on the collective opinion. Based on the input, the responses were taken and aggregated again. After *f* iterations, the process converges toward consensus, where the variance in expert responses decreases. The final set of aggregated responses is given as $A_f.$ The summary of the Delphi process can be written as a $f*N$ matrix computed using Eq. (2).

2 $$\begin{aligned} \begin{bmatrix} A_{1} \\ \vdots \\ A_{f} \end{bmatrix} = \begin{bmatrix} Q_{11} & Q_{12} & \cdots & Q_{1N} \\ \vdots & \vdots & \ddots & \vdots \\ Q_{f1} & Q_{f2} & \cdots & Q_{fn} \end{bmatrix} \end{aligned}$$

The approach used in this study defines consensus as a condition when variance is present. $\sigma ^2$ of response decreases over time.

3 $$\begin{aligned} \sigma ^2(r+1)<\sigma ^2(r) \end{aligned}$$

If the variance or disagreement among experts reaches a predefined threshold, we conclude that consensus has been reached, and the iterative process stops^25^. This process helps bring the group closer to consensus.

4 $$\begin{aligned} \sigma ^2(f)<\epsilon \end{aligned}$$

The Delphi method in this study involved four structured rounds of expert consultation, aimed at identifying and refining the key factors influencing the sustainability of LLM supply chains. In each round, a panel of ten experts with over ten years of experience in the AI and IT sectors provided open-ended responses to questions regarding sustainability drivers in LLM development and deployment. These responses were aggregated and anonymized after each round to avoid bias and were subsequently shared with the panel for further reflection and refinement.

The process emphasized iterative convergence: in Round 1, eight distinct factors were identified based on expert responses. In Rounds 2 and 3, these factors were discussed, and their overlaps were systematically analysed by the research team. Merging decisions were based on thematic similarity, expert feedback indicating conceptual redundancy, and clustering of related sustainability dimensions. For example, “Energy and Water Consumption” and “Environmental Impact of Hardware” were integrated into the broader category “Environmental Impact,” while “Ethical Considerations” and “Data Quality” were merged under “Data Quality and Ethical Considerations.

In Round 4, a consensus threshold of $\ge$ 65% agreement was applied to finalize the consolidation. This process resulted in four aggregated categories: (1) Environmental Impact, (2) Computational Efficiency and Resource Optimization, (3) Data Quality and Ethical Considerations, and (4) Social Responsibility and Governance. These categories were then used as the basis for the conjoint analysis.

### Analysis of utility

This study utilizes Ordinary Least Square (OLS) regression to estimate part-worth utilities and provide insights into consumer preferences and the relative importance of factors affecting the sustainability of LLMs supply chain. Conjoint analysis is a statistical technique used to determine how respondents value different factors of a supply chain of LLMs. It allows us to quantify respondents’ preferences by estimating the part-worth utilities associated with different attribute levels, which are used to evaluate the relative importance of product attributes. This study involved *N* respondents evaluating *J* profiles having different combinations of *K* factor, where each factor has *l* levels; the study used a fractional factorial design to ensure that all combinations are covered adequately. Respondents rank these profiles based on their preferences, allowing us to estimate part-worth utilities for each factor level. The utility $U_{ij}$ is that respondent *i* associate with profile *J* is given by a linear additive model given by the Eq. (5).

5 $$\begin{aligned} U_{ij}=\sum _{k=1}^{K}\beta _{ik}X_{jk}+\epsilon _{ij} \end{aligned}$$

Where, $\beta _{ik}$ represents the part-worth utility respondent *i* assigns to factor *k*, $X_{jk}$ is the dummy coded variable representing the level of the factor *k* in profile *J*. $\epsilon _{ij}$ is the error term accounting for unobserved factors.

Assuming that $\epsilon _{ij}$ following a Type I Extreme Value (Gumbel) distribution, this study estimates the utility function using ordinary least squares (OLS) regression for ranking data.

6 $$\begin{aligned} U_{ij}=\sum _{k=1}^{K}\beta _{k}X_{jk}+\epsilon _{ij} \end{aligned}$$

where $\beta _{k}$ represents the average part-worth utility of the factor *k* across all respondents. The OLS estimate of $\beta$ minimizes the sum of squared errors.

7 $$\begin{aligned} \widehat{\beta }=\arg {\min _{\beta }}\sum _{i=1}^{N}\sum _{j=1}^{J}\biggl (U_{ij}-\sum _{k=1}^{K}\beta _{k}X_{jk}+\epsilon _{ij} \biggr )^2 \end{aligned}$$

The relative importance $RI_k$ of factor is calculated by comparing the range of part-worth utilities across its levels to the total utility range across all attributes given by the Eq. (8).

8 $$\begin{aligned} RI_{k}=\frac{\max (\beta _{k})-\min _({\beta _k})}{\sum _{k=1}^{K}(\max (\beta _{k})-\min _({\beta _k}))}\times 100 \end{aligned}$$

The model validation uses holdout profiles that are not included in the estimation process. The study calculates predicted utilities for these holdout profiles and assesses model fit using measures such as hit rate, predictive accuracy, and $R^2$ for OLS models. The algorithm for the Conjoint Analysis approach used in this study is given in Algorithm 1.

**Algorithm 1:**
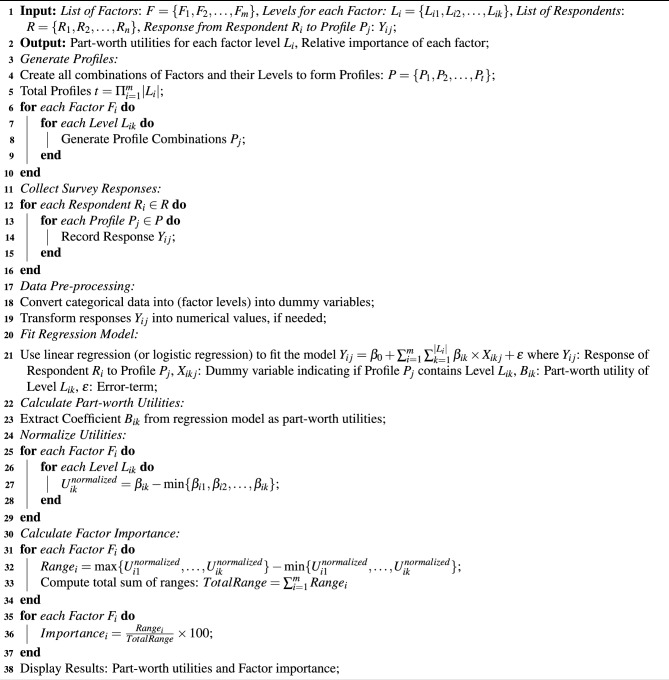
Conjoint Analysis

*Time complexity*: Profile generation in Steps 2-10 producing a complexity of $\mathscr {O}(t)=\Pi _{i=1}^m|L_i|$. Steps 11-16 for *R* respondents and *P* profiles shows the complexity of $\mathscr {O}(n.t)$. Further, the intervening Steps 17-19 are computed with a time complexity of $\mathscr {O}(1)$. Steps 20-21 perform model fitting with time-complexity of $\mathscr {O}(n.t.m)$. The rest of the steps execute with a time complexity of $\mathscr {O}(1)$. Therefore, the total time complexity for conjoint analysis algorithm is $\mathscr {O}(n^2t^3m)$.

### Sampling plan

The study uses purposive sampling to select the experts for the Delphi Study. Ten experts with more than ten years of experience in the information technology (IT) industry were interviewed to determine factors affecting the sustainability of the LLMs supply chain. The importance of these factors was calculated using a survey of a larger set of respondents working in the IT industry. (All methods were carried out in accordance with relevant guidelines and regulations. The study was reviewed and approved by the Institutional Review Board (IRB) of Gulf Medical University, Ajman, UAE. Informed consent was obtained from all participants prior to their participation in the study.) It was ensured that twenty respondents per profile were selected. At the same time, it was ensured that a minimum of 300 responses were taken as recommended in the extant literature^26^. Orthogonal Design of the profile was done using SPSS Software (IBM SPSS version 31.0.0.0. More details are available at https://www.ibm.com/products/spss-statistics). A total of 16 orthogonal profiles (out of a total of 81 profiles) were constructed using SPSS ORTHOPLAN based on a fractional factorial design. This design ensures statistical efficiency by minimizing redundancy while capturing the main effects across all factor levels. Each profile consisted of a unique combination of the four final factors (Environmental Impact, Computational Efficiency, Data Quality & Ethics, and Governance), with three levels per factor (Low, Medium, High). Profiles were randomly assigned and distributed electronically via email using a structured online survey tool to participants working in the IT and AI domains in Southeast Asia, the Middle East, and USA. The number of profiles presented to respondents was sixteen (Profile Number 1 to 16) for ranking, while the number of holdout cases was five (Profile Number 17 to 21), completely utilized for validation. The subjects judge holdout cases but are not used when the conjunction procedure estimates utilities. The response for the Conjoint Analysis was selected using purposive sampling. The profile was shared with more than 500 respondents working in the IT industry through email, out of which 314 responded, making it a 63 % response rate.

Sample Size Robustness (N = 314): The final dataset comprised 314 complete responses across 16 profiles. According to best practices in conjoint analysis, a minimum of 20 responses per profile is considered acceptable for reliability and model estimation. The data used in this study yields nearly 20 responses per profile per level combination, ensuring statistical adequacy and robustness for estimating part-worth utilities and calculating factor importance scores.

#### Response rate and data quality handling

The reported 63% response rate reflects strong engagement for an email-based survey in a professional population. The study included only complete and internally consistent responses in the analysis. Responses with missing rankings or inconsistent patterns were excluded during the preprocessing stage. To ensure the reliability and integrity of the data used in the conjoint analysis, several preprocessing and quality control measures were undertaken. First, only fully completed and internally consistent questionnaires were included in the final dataset. Responses exhibiting missing values, duplicate rankings, or illogical sequencing patterns were excluded during the preprocessing phase. Although traditional outlier detection techniques are not directly applicable to rank-order data, responses were screened for extreme or uniform patterns (e.g., ranking all profiles identically) that could bias utility estimations. Any such responses were removed before model estimation. To assess the predictive validity of the conjoint model, a holdout validation was performed using five profiles not included in the training phase. The agreement between predicted and actual rankings for these holdout profiles was evaluated using Kendall’s Tau, which yielded a value of 0.800 (p < 0.05), indicating strong alignment and confirming the internal validity and robustness of the model. These measures ensured that the input data were of high quality and suitable for generating reliable utility estimates and factor importance scores.

## Results

**Fig. 2:**
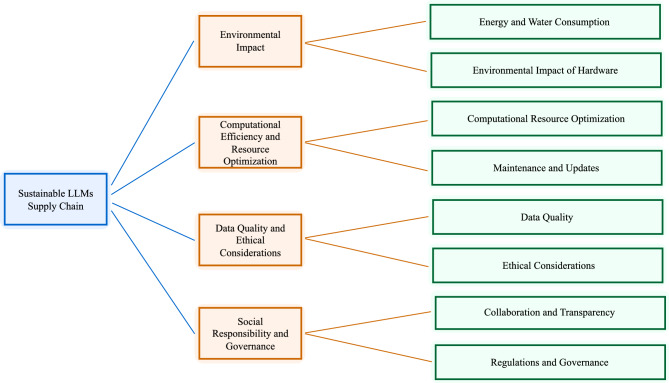
Hierarchical representation of the problem.

The Delphi Method was employed to determine the factors affecting the sustainability of the LLMs supply chain. The Delphi process lasted four rounds, and the number of factors was reduced from eight to four. The factors that overlap were combined under four categories, finally taken as four factors to be considered for the conjunction analysis. The results of the Delphi Process are listed in Fig. 2.

The researcher involved in the study removed one factor as it overlaps with the existing factors. It was done because one of the requirements of the conjoint is attributed to the profile being distinct from each other. All factors except the Environmental Impact (F1) are assumed to increase linearly in the conjoint model. The correlations between observed and estimated preferences for the Conjoint Analysis are given in Table 2.

**Table 2:** Correlation matrix for the conjoint analysis.

	Value	Sig.
Pearson’s R	.438	.045
Kendall’s Tau	.310	.048
Kendall’s Tau for Holdouts	.800	.025

Pearson’s R (0.438) indicates a moderate positive correlation between the observed and predicted preferences, demonstrating a reasonable fit between respondents’ actual preferences and the model’s estimations. This suggests that while the model captures the general trend, individual variations exist. Kendall’s Tau (0.310) further confirms moderate agreement between respondents’ actual rankings and the model’s predicted rankings, reflecting reliability in ordinal decision-making about factor importance. Kendall’s Tau for Holdouts (0.800) highlights robust predictive accuracy, indicating the model’s strong potential for generalization to similar contexts or larger populations. Holdout validation was performed using five profiles not included in the model training phase. Kendall’s Tau for holdouts was calculated as 0.800 (p < 0.05), indicating a strong alignment between predicted and actual respondent preferences.

**Table 3:** Utility estimates for different factors.

		Utility estimate	Std. error
F1	Low	.227	1.486
	Medium	.453	2.972
	High	.680	4.459
F2	Low	-.425	1.486
	Medium	-.850	2.972
	High	-1.274	4.459
F3	Low	2.267	1.486
	Medium	4.533	2.972
	High	6.800	4.459
F4	Low	.637	1.486
	Medium	1.274	2.972
	High	1.911	4.459
(Constant)		3.766	5.346

**Fig. 3:**
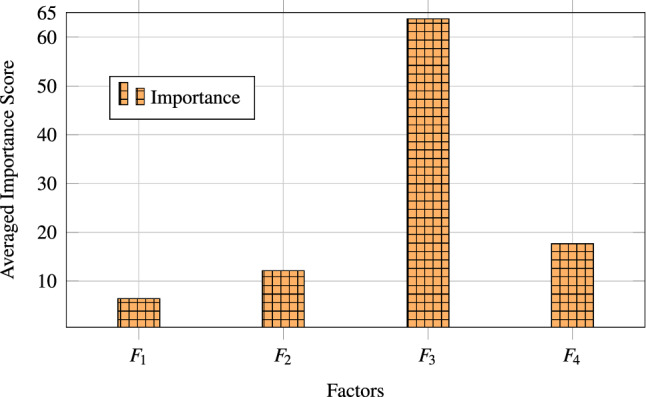
Averaged importance score of factors affecting sustainability.

As elaborated in Table 3 the result of the study suggests that environmental impact (F1) gets a greater utility estimate for high levels of the factor. This is surprising as it suggests that respondents are less concerned about the environmental impact of the LLMs supply chain. Similar results were observed for Computational Efficiency and Resource Optimization (F2) as the higher level witnesses, the lower utility for Data Quality and Ethical Considerations (F3) and Social Responsibility and Governance (F4) utility increases if the supply chain performance is improved for these factors. The result shows that data quality, ethical considerations (F3), and social responsibility and governance (F4) have higher utility for respondents than the other two factors. The study then calculated the average importance score for the four factors as F1(6.488), F2(12.123), F3( 63.653), and F4 (17.735) as depicted in Fig. 3.

## Discussions

The results of the study are alarming. The study found that respondents are less concerned about the environmental impact of LLMs, such as energy consumption, water consumption, e-waste, computational efficiency, and resource optimization. The study contradicts a recent study that raised concerns about the increase in LLMs’ life-cycle energy and carbon footprints^27^. The priorities expressed by respondents through conjoint analysis differed notably from the importance of sustainability highlighted in the existing literature, reflecting potential gaps in awareness or immediate prioritization. This study concludes that there is either ignorance or lack of awareness about the carbon footprint of the LLMs’ supply chain. Another extant study highlights the impact of the cloud-based servers and concerns related to water consumption^28^. The respondents of this study have less utility for such environmental concerns. The respondents of this study seem to be less concerned about hidden ecological costs, although they give some preference to ethical and legal concerns. Extant literature warns against hidden ecological cost of cloud computing^29^. The study’s respondents show higher utility for Data Quality and Ethical Considerations. This finding concurs with a recent article published in Nature^30^. Social Responsibility and Governance are important factors contributing to the sustainability of the LLMs supply chain. This study’s findings align with a recent study published^31^. Thus, this study acts as an eye-opener for the AI Governance Authorities.

The low utility of environmental impact and resource optimization suggests that people using and creating LLMs daily put less importance on this important consideration. Thus, the role of government becomes very important in creating guidelines on the environmental impact of the LLMs. The efforts of Information Technology to consider environmental impact minimization under corporate social responsibility is another idea. For example, these companies that use or create a large language model should invest some of their profit in tree implantation and rainwater harvesting. Another idea may be placing the servers used in LLMs supply chain geographically, where they are powered by green energy. However, these measures will affect data sovereignty, an important requirement for LLMs in industries such as healthcare. Federated Learning offers a trans-formative approach to resolving the challenges posed by data sovereignty^32,33^. Keeping data local and only sharing encrypted model updates enables organizations to collaborate across borders while ensuring compliance with local regulations. These ideas will only be seen in light if there is awareness about the factors affecting the sustainability of LLM’s supply chain. Government agencies should audit Information Technology companies for their sustainability initiatives.

To mitigate the environmental footprint of LLMs, several emerging solutions are being implemented. These include the use of low-energy training algorithms like sparse training, model pruning, and knowledge distillation, which significantly reduce computational overhead. In parallel, leading AI firms are migrating training infrastructure to renewable energy-powered data centers, such as those run on solar or hydropower. Hardware innovations also play a vital role application-specific integrated circuits (ASICs) and field-programmable gate arrays (FPGAs) offer considerable energy efficiency compared to general-purpose GPUs. Moreover, model quantization techniques enable reduced-precision training with minimal accuracy loss, thereby reducing power consumption.

Stakeholder collaboration is essential for ensuring the sustainability of LLM supply chains. Governments play a regulatory and incentivizing role by enforcing carbon accounting, mandating sustainability disclosures, and supporting green AI infrastructure through subsidies or public-private partnerships^34^. Cloud providers such as AWS, Azure, and Google Cloud have a pivotal role in energy optimization, transitioning to renewable energy sources, and offering model training APIs with transparency around carbon footprint metrics. Researchers contribute by developing energy-efficient model architectures, exploring low-resource fine-tuning techniques, and proposing policy frameworks for ethical AI governance. Effective collaboration among these entities can accelerate the shift toward environmentally responsible and socially equitable AI ecosystems.

The relatively low importance score assigned to the Environmental Impact factor (6.488) may stem from several underlying causes. First, there exists a notable gap in awareness and accessibility of environmental impact data related to LLM development, particularly among practitioners in non-academic or industry-focused roles. Second, the prevailing emphasis on performance, cost-efficiency, and rapid deployment in the AI industry often overshadows long-term sustainability goals. Third, in many developing or transitional economies, environmental sustainability metrics are seldom incorporated into procurement criteria or operational KPIs, resulting in their marginal prioritization. These findings are consistent with the phenomenon of environmental myopia in technology adoption, where immediate functional benefits outweigh ecological considerations^29^. Future studies could further investigate these drivers through interviews or behavioral modeling.

While federated learning (FL) presents a promising solution for preserving data sovereignty by keeping data local, it is not without significant trade-offs. FL can introduce substantial computational burdens on edge devices, particularly in energy-constrained environments. Moreover, the frequent exchange of encrypted model updates across devices can result in high communication overhead and synchronization delays. These challenges are magnified in industries like healthcare or finance, where diverse data types, regulatory compliance, and device interoperability issues complicate deployment. Therefore, FL should be approached as a complementary rather than a universal solution to sustainability and data sovereignty in LLM supply chains.

The sustainability of LLM supply chains aligns closely with several United Nations Sustainable Development Goals (SDGs). Specifically, SDG 9 (Industry, Innovation, and Infrastructure) is addressed through the promotion of efficient AI infrastructure and responsible innovation. SDG 12 (Responsible Consumption and Production) is relevant in minimizing energy consumption, e-waste, and optimizing resource use throughout the LLM lifecycle. Moreover, SDG 13 (Climate Action) is indirectly supported by advocating for carbon-conscious model training and deployment practices. By integrating sustainability considerations into AI development pipelines, the LLM ecosystem can contribute to global Environmental, Social and Governance (ESG) objectives.

The prioritization of ”Social Responsibility and Governance” by respondents may also reflect the increasing influence of evolving AI regulatory landscapes. Frameworks such as the European Union AI Act, the OECD AI Principles, and national AI governance strategies are pushing organizations to embed accountability, transparency, and ethical compliance into AI development and deployment processes^35^.

## Conclusions

The use of LLMs is increasing in every industry. This phenomenal growth has its advantages as well as disadvantages. It will help increase productivity and bring efficiency in many industries; on the other hand, its supply chain is being scrutinized for sustainable practices. The supply chain of LLMs involves multiple interconnected stages. It begins with specialized hardware, such as high-performance GPUs, TPUs, and chips essential for training and running these models. These components are hosted in large data centres operated by cloud providers. The development process involves training that requires massive datasets aggregated from public and proprietary sources. The energy demands for training and inference are substantial, highlighting environmental considerations. This supply chain depends heavily on global logistics, advanced hardware, skilled labour, and ethical data sourcing, ensuring LLMs are built and maintained efficiently.

The study has three implications for the practice. Firstly, it identifies the factors affecting the sustainability of the LLMs supply chain. The four categories of these factors were identified as Environmental Impact, Computational Efficiency & Resource Optimization, Data Quality & Ethical Considerations, Social Responsibility & Governance, and Infrastructure & Hardware Sustainability. Second, this study determines the utility of different factors and calculates the importance score for these identified factors. Finally, this study provides an approach to making the supply chain of LLMs sustainable. The study concludes that there is a lack of awareness about the environmental impact of LLMs. The factor Data Quality & Ethical Considerations gets the highest importance score. The study uses respondents from Southeast Asia, the Middle East and the USA, and a future study can compare the results for different geographic areas.

The finding that respondents assigned low importance to environmental concerns suggests a critical gap in awareness, likely influenced by the limited availability of environmental transparency from LLM vendors, the underrepresentation of sustainability in AI-related discourse, and the absence of environmental impact considerations in most AI training and development frameworks. This highlights the urgent need for increased transparency, inclusion of sustainability in professional education, and industry-wide discourse on the lifecycle impacts of LLM deployment. To improve the sustainability of LLM supply chains, organizations should adopt concrete strategies, including (a) reporting energy, carbon, and water metrics; (b) investing in efficient LLM architecture; (c) geographically optimizing server placement; (d) evaluating upstream suppliers based on environmental KPIs; and (e) participating in multi-stakeholder initiatives to co-create sustainability benchmarks.

The insights derived from this study offer a foundation for targeted policy interventions and practical strategies aimed at improving the sustainability of large language model (LLM) supply chains, particularly in resource-constrained settings. Federated learning approaches can play a crucial role by reducing reliance on centralized data centres, thereby decreasing overall energy consumption and simultaneously enhancing data sovereignty. In parallel, renewable energy–based training centres powered by solar or hydropower can be supported through government subsidies or public–private partnerships to mitigate the environmental footprint of model training. Moreover, governments and regulatory bodies can introduce mandatory AI sustainability audits that require standardized reporting on key indicators such as energy usage, carbon emissions, and water consumption. The promotion of energy-efficient AI architectures, including sparse training, model pruning, quantization, and knowledge distillation, should be incentivized to lower computational overhead without compromising model performance. Additionally, optimizing the geographic placement of servers and incorporating environmental performance criteria into the evaluation of upstream suppliers can further enhance the sustainability of LLM deployment frameworks. These recommendations are aligned with international standards such as the Green Software Foundation and ISO 14001 and collectively offer a pragmatic roadmap for balancing AI innovation with environmental stewardship and social responsibility.

### Limitations and future directions

Future efforts toward sustainable LLM supply chains should prioritize integration of green technologies, such as training with renewable energy and optimizing algorithmic efficiency. By combining advances in hardware efficiency, training compression, and data center sustainability, LLM deployment can align with global climate goals while ensuring responsible AI adoption in resource-constrained contexts. While this study provides valuable insights into the sustainability priorities within LLM supply chains, it has some inherent limitations. The use of purposive sampling, although adequate for exploratory research, may limit the broader applicability of findings. The preferences captured reflect stakeholder perceptions, which may vary with awareness and experience levels. Additionally, the expert panel, though experienced, was primarily composed of IT professionals, and perspectives from environmental scientists or policymakers could enrich future analysis. The study focused on qualitative prioritization and did not integrate detailed quantitative metrics such as actual energy or carbon usage. Future research could expand stakeholder diversity, incorporate lifecycle environmental data, and explore region-specific differences. Developing a comprehensive sustainability index and validating findings through case studies or behavioral experiments would further enhance the robustness and practical utility of this research in shaping responsible AI development. While our sample included respondents from Southeast Asia, the Middle East, and the United States, this study did not conduct a comparative regional analysis. Differences in sustainability priorities across geographies may exist due to regulatory frameworks, resource availability, or cultural factors. Future studies could explore this dimension through region-specific sampling and cross-tabulation of responses, providing a more granular understanding of contextual variations in factor importance.

## Data Availability

The orthogonal plan, ranking data, and syntax used for the conjoint analysis are available in .sav format in a public repository at DOI: 10.6084/m9.figshare.27282939. Additionally, the plan cards (profiles) and data are provided in both MS Word and MS Excel formats in the same repository (https://figshare.com/articles/dataset/Conjoint_Design_Syntax_and_Profiles/27282939). This ensures accessibility and transparency for replication and further research.

## Declarations

### Software Details

Orthogonal Design of the profile was done using SPSS Software IBM SPSS version 31.0.0.0. More details regarding this are available at https://www.ibm.com/products/spss-statistics.
